# The Formation Mechanism and Corrosion Resistance of a Composite Phosphate Conversion Film on AM60 Alloy

**DOI:** 10.3390/ma11030402

**Published:** 2018-03-08

**Authors:** Jun Chen, Xiangna Lan, Chao Wang, Qinyong Zhang

**Affiliations:** 1 Key Laboratory of Fluid and Power Machinery of Ministry of Education, School of Materials Science and Engineering, Xihua University, Chengdu 610039, China; veralxn@126.com (X.L.); bohr123@163.com (Q.Z.); 2 Clean Energy Materials and Engineering Center, School of Electronic Science and Engineering, State Key Laboratory of Electronic Thin Film and Integrated Devices, University of Electronic Science and Technology of China, Chengdu 611731, China; cwang@uestc.edu.cn

**Keywords:** AM60 magnesium alloy, phosphate, chemical composition analysis, corrosion

## Abstract

Magnesium alloy AM60 has high duc and toughness, which is expected to increase in demand for automotive applications. However, it is too active, and coatings have been extensively studied to prevent corrosion. In this work, a Ba-containing composite phosphate film has been prepared on the surface of AM60. The composition and formation mechanism of the film have been investigated using a scanning electronic microscope equipped with energy dispersive X-ray spectroscopy, Fourier transform infrared, X-ray photoelectron spectroscopy, and X-ray diffractometry tests. The corrosion resistance of the film has been measured by electrochemical and immersion tests. The results show that the deposition film has fully covered the substrate but there are some micro-cracks. The structure of the film is complex, and consists of MgHPO_4_·3H_2_O, MnHPO_4_·2.25H_2_O, BaHPO_4_·3H_2_O, BaMg_2_(PO_4_)_2_, Mg_3_(PO_4_)_2_·22H_2_O, Ca_3_(PO_4_)_2_·xH_2_O, and some amorphous phases. The composite phosphate film has better anticorrosion performance than the AM60 and can protect the bare alloy from corrosion for more than 12 h in 0.6 M NaCl.

## 1. Introduction

With excellent properties, such as low density, mechanical stability, and high damping capacity, magnesium (Mg) alloys are attracting much recent attention. However, Mg alloys have not been widely used yet due to the poor corrosion resistance, which is the main undesirable property [1]. Surface treatment is a general way to control corrosion by forming a barrier layer to isolate the bare alloys from the environment [2]. Phosphate conversion coatings (PCCs) are promising coatings because most metal phosphates are insoluble in water and have high chemical stability [3]. The utilization of PCCs has a history of centuries, and this traditional mature technology has been successfully exploited to protect steel, zinc, and aluminum [4,5,6]. 

PCCs on Mg alloys have been investigated widely [7,8,9,10,11,12,13,14,15,16,17,18,19]. Phosphating has always been carried out in the acidic solution containing Mn^2+^, Zn^2+^, Ca^2+^, Na^+^, and Mg^2+^ [13]. For example, Phuong et al. synthesized a Zn PCC on AZ91, which consisted of an outer crystal Zn_3_(PO_4_)_2_·4H_2_O layer and inner MgZn_2_(PO_4_)_2_ and Mg_3_(PO_4_)_2_ layer. The longest corrosion initiation time of the coated sample was about 12 h in 0.5 M NaCl solution [7]. Song et al. has improved the generally porous structure of the Ca PCCs through an environmentally friendly solution containing Ca(NO_3_)_2_ and NH_4_H_2_PO_4_ with ultrasonic agitation. However, the dissolution of the flake particles into small chipping occurred after being immersed in the simulated body fluid for 2 h [9]. Zhou et al. prepared a Mn PCC, the corrosion potential (*E*_corr_) and the radius of the capacity impedance of which increased distinctly in 3.5 wt % NaCl solution compared with that of the AZ91 substrate, displaying excellent anticorrosion performance [8]. Chen et al. [12] pointed out that Mn PCC had desirable corrosion resistance, which was more stable and corrosion resistant than its Zn and Ca peers. Recently, Ba PCC has been developed for the corrosion protection of Mg alloys [14,15,16], which also shows great anticorrosion properties. For instance, it was reported that the coating prepared in a simple solution with Ba(NO_3_)_2_ and NH_4_H_2_PO_4_ could effectively decrease the corrosion current density (*i*_corr_) of the AZ31 alloy from 154 to 3.78 μA·cm^−2^ in 5 wt % NaCl solution [15]. In addition, the combination of different phosphates has a tendency to strengthen the corrosion resistance of the coatings. Wang et al. synthesized a Zn–Mn PCC, which was composed of Zn, Zn_3_(PO_4_)_2_, MnHPO_4_, and Mn_3_(PO_4_)_2_. The corrosion properties of Mg–Li alloy were improved greatly by this composite coating [11]. Hence, in the present work, we choose Mn^2+^ and Ba^2+^ as the main ingredients in order to obtain an anticorrosive coating. However, the Ba PCC prepared by Chen [15] featured a two-layer structure, and the top layer consisted of large crystals exhibiting a lower adhesion than the under layer. Therefore, further studies are necessary to improve the present process. Zhou et al. pointed out that adding a Ca^2+^ compound in the bath could improve the combination between the substrate and coating [18]. In order to achieve both high corrosion resistance and strong adhesion, a small amount of Ca(NO_3_)_2_ is also added in this work.

Moreover, it is notable that the composition of the PCCs prepared in solutions containing various anions is always complicated. In the previous work, Liu et al. prepared a Ba PCC on AZ91D Mg alloy with Ba(NO_3_)_2_, Mn(NO_3_)_2_, and NH_4_H_2_PO_4_ as precursors [16]. However, the component of the coating was only tested using the energy dispersive X-ray spectroscopy (EDS), and the real composition of the coating was unclear. It is also remarkable that the phosphating mechanism may vary in different phosphating systems. As a result, this work aims at clearly illustrating the structure and formation mechanism of the coating prepared using the present process. The corrosion performance of the coating is also studied.

## 2. Experimental

### 2.1. Fabrication of the Film

The material used in this study was die-cast AM60 alloy (5.94 wt % Al, 0.43 wt % Mn, 0.11 wt % Zn, 0.03 wt % Si, 0.004 wt % Cu, 0.001 wt % Fe, and bal. Mg). The samples were ground with 2000 grit SiC paper, ultrasonically cleaned in ethyl alcohol, and then dried in the cold air. According to our preliminary experiment exploration, the optimum formation process has been chosen to synthesize the film, which is as follows. 10 mL·L^−1^ Mn(NO_3_)_2_, 10 g·L^−1^ Ba(NO_3_)_2_, 6 g·L^−1^ Ca(NO_3_)_2_·4H_2_O, and 20 g·L^−1^ NH_4_H_2_PO_4_ were selected as the ingredients of the treating solution. The pH value of the solution was settled to 3, the treating temperature was room temperature (RT) (30 ± 2 °C), and the treating time was 0.5 h. Then, in order to compare the adhesion of the film formed by the above process with an appropriate concentration of the Ba^2+^ ingredient, another film was prepared at 60 °C in the solution containing of 10 mL·L^−1^ Mn(NO_3_)_2_, 15 g·L^−1^ Ba(NO_3_)_2_, and 20 g·L^−1^ NH_4_H_2_PO_4_, which was named as the film for comparison.

### 2.2. Characterization

The morphology of the film was observed using a Quanta 250 FEG environmental scanning electronic microscope (ESEM, FEI, Hillsboro, OR, USA). The composition was analyzed by EDS, Fourier transform infrared (FTIR), X-ray photoelectron spectroscopy (XPS) and X-ray diffraction (XRD). The FTIR spectrum was obtained on a Tensor 27 spectrometer (Bruker, Karlsruhe, Germany) in the wavenumber range of 400–4000 cm^−1^. The XPS was probed using an ESCALAB 250 XPS (Thermo Fisher Scientific, Waltham, MA, USA) with Al K_α_ radiation. The power was 150 W, the pass energy was 50.0 eV, and the step size was 0.1 eV. The energy values were referenced to the adventitious C1s peak at 284.6 eV. XRD was carried out on a PW1700 diffractometer (Philips, Amsterdam, The Netherlands) with a Cu target (λ = 0.154 nm). The film was scraped from the samples and prepared as the finely pressed powderfor the XRD test. Electrochemical tests were performed using a ParStat 4000 potentiostat (Ametec, Berwyn, PA, USA) with a three-electrode cell system, which consists of a saturated calomel electrode (SCE) reference electrode, a platinum counter electrode, and a working electrode with an exposed area of 1 cm^2^. The samples were immersed in the aggressive medium for 300 s before the experiment. The polarization curves were obtained at a constant voltage scan rate of 0.5 mV·s^−1^. Electrochemical impedance spectroscopy (EIS) was conducted with the frequency swept from 100 kHz to 10 mHz, with a perturbation amplitude of 5 mV. An immersion test was carried out according to GB 10124-88 of China. The corrosion testing solution was 0.6 M NaCl solution at RT.

## 3. Results and Discussion

### 3.1. SEM Morphology and Adhesion of the Films

**Figure 1:**
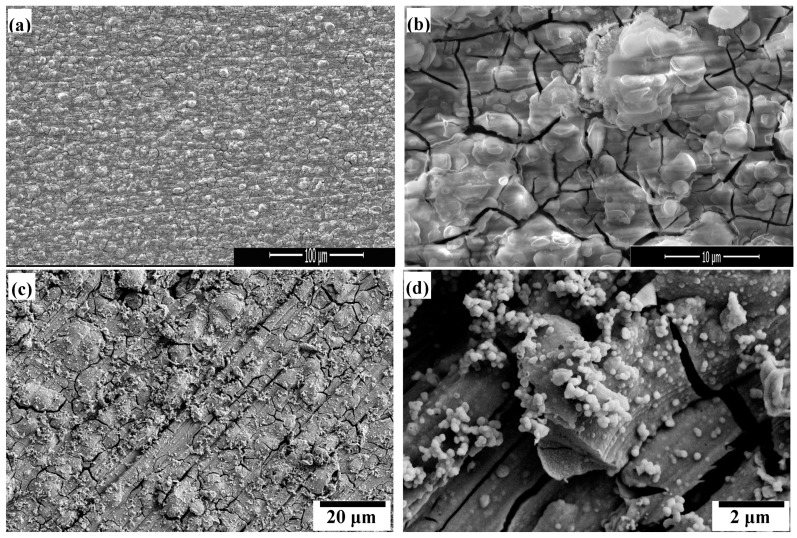
SEM morphology of different films: (**a**,**b**) the film formed by the optimum process and (**c**,**d**) the film for comparison.

The SEM morphology of the films is shown in Figure 1. The deposition film prepared by the optimum formation process has fully covered the substrate, but micro-cracks can also be observed. The surface of the film is not very smooth and has tubercles. Many island particles can be recognized from the high-resolution image. Cracks are frequently observed in PCCs on Mg alloys. These cracks might arise from the severe H_2_ evolution during the synthesis process and the dehydration. During the drying process, the coexistence of voids caused by H_2_O evaporation and residual stress in the coating led to the coating shrinkage and crack formation [15,20,21,22,23,24]. As a protective coating, such a surface cannot provide long-term protection because of the cracked microstructure [23]. However, the cracking of the surface is hard to avoid, thus the protective effect of the PCCs may be limited. In Figure 1c,d, the surface of the film for comparison formed in the solution with high Ba^2+^ concentration and no Ca^2+^ ingredient was not very “clean”, which was much rougher compared to the film in Figure 1a. From the high-resolution image, it is observed that the film is heterogeneous with a combination of nanosphere particles. These particles may be easily detached, resulting in the poor adhesion of the film. As a consequence, the film prepared by the optimum formation process exhibits better adhesion than the film for comparison, according to a simple tape test (i.e., the adhesion of the tape is worse after it is pulled off from the film for comparison). 

### 3.2. Composition Analysis of the Film

The chemical composition of the film was analyzed using EDS, FTIR, XPS, and XRD. The content of various elements of the film detected by EDS is displayed in Table 1. It discloses that the composition of the film is very complex, including C, O, P, Mg, Ca, Mn, and Ba. The content of Ca is much less than that of the other metal elements. It should be mentioned that the signal of Al may be mainly attributed to the matrix, because Al has not been tested by the XPS analysis.

**Figure 2:**
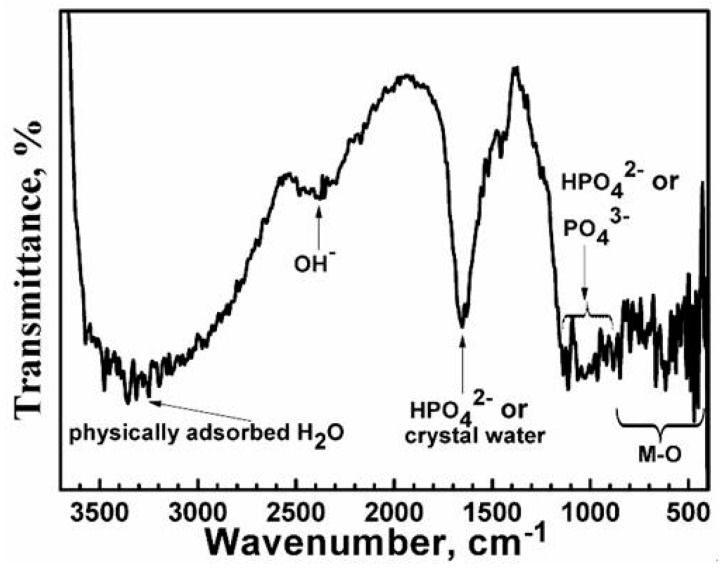
FTIR spectrum of the film.

The FTIR spectrum of the film is shown in Figure 2. Physically adsorbed water molecules can be identified by the broad band around 3100–3500 cm^−1^ [25,26]. The band at 2380 cm^−1^ may correspond to the vibration of OH^−^, and the strong peak around 1658 cm^−1^ may be ascribed to HPO_4_^2−^ or crystal water in the film [19,27]. In addition, many peaks are observed in the range of 830–1140 cm^−1^, which may be assigned to the vibration band of PO_4_^3−^ or HPO_4_^2−^ [19,27,28,29,30]. Furthermore, it can be seen that the peak for HPO_4_^2^^−^ is much sharper, indicating that the content of the HPO_4_^2−^ anions is much higher than PO_4_^3−^. The bands in the range of 400–800 cm^−1^ are attributed to metal–oxygen stretching [31,32]. Because there are too many kinds of metal ions, the peaks in this range are very complex. 

**Figure 3:**
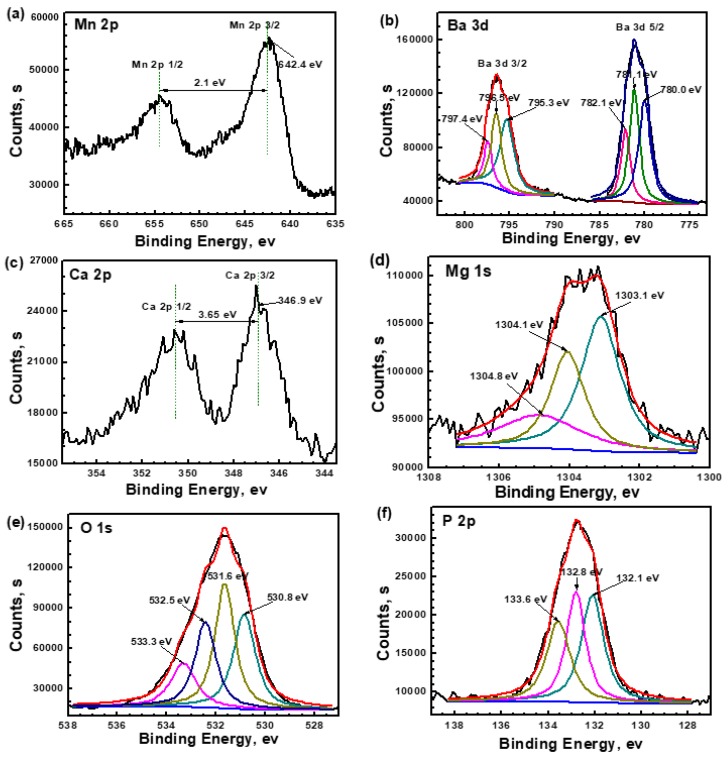
XPS analysis of the film after 30 s etching: (**a**) Mn 2p; (**b**) Ba 3d; (**c**) Ca 2p; (**d**) Mg 1s; (**e**) O 1s; and (**f**) P 2p.

**Figure 4:**
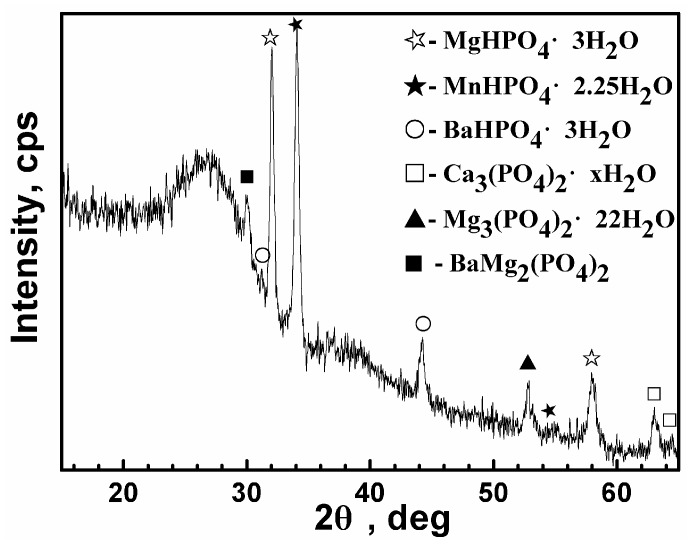
XRD spectrum of the film.

Figure 3 shows the XPS analysis of the film after 30 s of etching. The high-resolution spectra of Mn 2p, Ba 3d, and Ca 2p all show two distinctive peaks as a result of spin orbit splitting, which is the energy peak at lower energy and is the satellite peak at high energy. The peak corresponding to Mn 2p_3/2_ is located at 642.4 eV in Figure 4a. The binding energy (BE) distance between the energy peak and satellite peak is 2.1 eV. The Mn 2p_3/2_ peak shifts toward the higher BE compared to that of Mn_3_(PO_4_)_2_ in the reference of [12]. Hence, the composition of Mn^2+^ may be assigned to MnHPO_4_. Figure 4b shows two distinctive peaks, Ba 3d_5/2_ and Ba 3d_3/2_, which are both divided into three separate peaks. The width of three pairs of Ba 3d peaks is about 15.3 eV, which can be attributed to Ba^2+^ [15,33]. The peak of Ba 3d_5/2_ at 780.0 eV can be attributed to BaHPO_4_ [15]. The BE of the peak at 782.1 eV is a little larger than that of Ba_3_(PO_4_)_2_ in the reference [15], indicating that this component is not a simple Ba_3_(PO_4_)_2_, and the Ba–PO_4_ bond may be bonded with other elements to form a more complicated component. There is an intensive peak at 781.1 eV, indicating another Ba^2+^ compound. However, there is no material related to this BE in the existing database. The high-resolution spectrum of Ca 2p splits into two peaks, Ca 2p_3/2_ and Ca 2p_1/2_, with a separation of about 3.65 eV. The peak of Ca 2p_3/2_ at 346.9 eV can be attributed to tricalcium phosphate Ca_3_(PO_4_)_2_ [34]. The Mg 1s spectrum is broad, which can be resolved into three components. BE at 1303.1 eV is attributed to MgHPO_4_ [24,35]. The peak at 1304.1 eV is assigned to Mg_3_(PO_4_)_2_ [19]. There is a small shoulder peak at the high BE 1304.8 eV, indicating another Mg^2+^ compound, but the exact component is difficult to identify due to the absence of more reliable data. Figure 4e presents the high resolution spectrum of O 1s, which is deconvoluted into four peaks. The peak at 533.3 eV can be attributed to P–OH [24,35]. The BE at 532.5 eV is H_2_O, but it should be in the form of crystallization water [36]. The peak at 531.6 eV is attributed to P=O, while the peak at 530.8 eV may be attributed to P=O or OH^−^ [15,24,35]. The spectrum of P 2p is divided into three peaks, 132.1, 132.8, and 133.6 eV. The peak at low BE is assigned to PO_4_^3−^, while the other two higher BE peaks are attributed to HPO_4_^2−^ [12,19,24,37,38]. It can be implied that hydrogen phosphates are the main components and phosphates are the minor components. This result is in accordance with the FTIR detection that the content of HPO_4_^2−^ is much more than that of PO_4_^3−^. 

To further confirm the structure of the film, XRD measurement was also carried out as shown in Figure 4. The major phases in the film are hydrophosphates MgHPO_4_·3H_2_O, MnHPO_4_·2.25H_2_O, and BaHPO_4_·3H_2_O, as well as a minority of three other phosphates, including BaMg_2_(PO_4_)_2_, Mg_3_(PO_4_)_2_·22H_2_O, and Ca_3_(PO_4_)_2_·xH_2_O. It is in accordance with the FTIR and XPS analysis that the main anion in the film is HPO_4_^2−^. The content of Ca_3_(PO_4_)_2_·xH_2_O is small, which is in accordance with the EDS analysis that the content of Ca is much less than that of the other metal elements. The phases in the film tested by XRD are coincident with the XPS analysis. It is noticed that there is an unknown peak in the XPS spectrum of both Ba 3d and Mg 1s, which may correspond to this complicated component, BaMg_2_(PO_4_)_2_. In addition, a broad diffuse diffraction pattern located at 2θ = 23°–29° is observed, indicating that the film is composed of some amorphous phases. Regarding the phenomenon where there was a small content of BaHPO_4_·3H_2_O and BaMg_2_(PO_4_)_2_ tested by XRD but there was a great amount of Ba element detected by EDS, it suggests that the amorphous phases may be mainly Ba-containing compounds. The XRD pattern in other works has also confirmed the lack of crystallinity in the Ba phosphate cement [14]. Hence, it can be implied that the intensive Ba 3d_5/2_ peak at 781.1 eV may be assigned to the amorphous compound. In other words, XRD shows both the amorphous and crystalline nature of this composite PCC, and the crystalline phases are hydrophosphates or phosphate compounds. 

On the basis of the above composition analysis, an analysis of the formation mechanism of this composite phosphate film has been proposed. The possible reactions are listed as follows.

Firstly, once the substrate is exposed to the phosphate electrolyte solution, Mg is dissolved to produce Mg^2+^, resulting in a large increase in the OH^−^ concentration and hydrogen evolution.

Mg → Mg^2+^ + 2e (1)

2H_2_O + 2e →H_2_ ↑ + 2OH^−^ (2)

In addition, the dissolution reaction is much faster in the acidic bath, resulting in the generation of more hydrogen and promoting the OH^−^ concentration at the interface of the metal and the solution. 

Subsequently, the OH^−^ reacts with H_2_PO_4_^−^ to form HPO_4_^2^^−^. As we know, H_2_PO_4_^−^ is easily reduced to HPO_4_^2−^, but HPO_4_^2−^ is difficult to reduce to PO_4_^3−^ because of the strong bond energy of H^+^ and PO_4_^3−^ [9]. In addition, the H_2_ evolution can resist further movement of HPO_4_^2^^−^ in the solution to the substrate surface [35]. As a result, only minor amounts of HPO_4_^2^^−^ in the solution adjacent to the metal surface continues to react with OH^−^ to form PO_4_^3^^−^, which corresponds to the low concentration of PO_4_^3−^ in the film. The thermodynamics data shows that the solubility–product constants (*K*_sp_) of Ca_3_(PO_4_)_2_, Mg_3_(PO_4_)_2_·8H_2_O, and Ba_3_(PO_4_)_2_ are 2.0 × 10^−29^, 6.3 × 10^−26^, and 3.4 × 10^−23^, respectively. Consequently, these minor PO_4_^3^^−^ ions preferentially bond with Ca^2+^ to form the more insoluble compound Ca_3_(PO_4_)_2_·xH_2_O, then Mg_3_(PO_4_)_2_. However, Ba_3_(PO_4_)_2_ is not contained in the mixture form of BaMg_2_(PO_4_)_2_. A similar phenomenon was observed in other mixture phosphates, e.g., calcium magnesium phosphates and Zn_2_Mg(PO_4_)_2_, which were mostly referred to as Mg-doped Ca or Zn phosphates [20,39]. The above analysis can be explained by the following reactions:

H_2_PO_4_^−^ + OH^−^ → HPO_4_^2^^−^ (3)

HPO_4_^2^^−^ + OH^−^ → PO_4_^3^^−^ (4)

3Ca^2^^+^ + 2PO_4_^2^^−^ + xH_2_O → Ca_3_(PO_4_)_2_·xH_2_O (5)

2Mg^2^^+^ + 3PO_4_^2^^−^ + 22H_2_O → Mg_3_(PO_4_)_2_·22H_2_O (6)

Ba^2+^ + 2Mg^2^^+^ + 2PO_4_^2^^−^ → BaMg_2_(PO_4_)_2_ (7)

Massive Mg^2+^ ions depart from the metal crystal lattice and diffuse towards the bulk solution. Mn^2+^ and Ba^2+^ in the solution diffuse towards the metal surface. The AM60 substrate is surrounded by a large number of HPO_4_^2^^−^ ions in the solution. Thus Mg^2+^, Mn^2+^, and Ba^2+^ encounter HPO_4_^2^^−^ to form MgHPO_4_·3H_2_O, MnHPO_4_·2.25H_2_O, and BaHPO_4_·3H_2_O, respectively.

Mg^2+^ + HPO_4_^2^^−^ + 3H_2_O → MgHPO_4_·3H_2_O (8)

Mn^2+^ + HPO_4_^2^^−^ + 2.25H_2_O → MnHPO_4_·2.25H_2_O (9)

Ba^2+^ + HPO_4_^2^^−^ + 3H_2_O → BaHPO_4_·3H_2_O (10)

Finally, with the existence of Mg^2+^ from the substrate, as well as Mn^2+^, Ca^2+^, and Ba^2+^ in the solution, complex insoluble phosphorous compounds corresponding to these cations are deposited on the surface of the AM60 alloy to form the multi-phase composite conversion film.

### 3.3. Corrosion Performance of the Film

**Figure 5:**
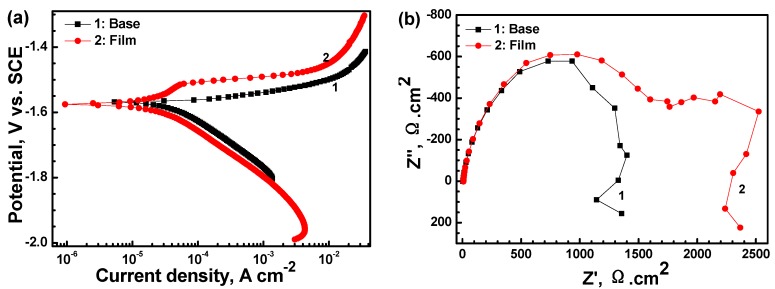
(**a**) Polarization curves and (**b**) Nyquist plots of the AM60 alloy with and without film immersed in 0.6 M NaCl solution.

**Figure 6:**
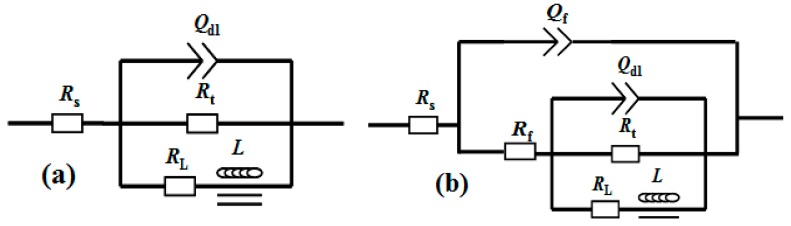
Equivalent circuits for the EIS spectra of: (**a**) the AM60 substrate and (**b**) the sample coated with film immersed in 0.6 M NaCl solution.

Figure 5 displays the electrochemical tests of the AM60 alloy with and without film in 0.6 M NaCl solution. Three samples were used for the polarization test. Only one polarization curve of the samples was shown, but the degree of uncertainty was given based on the three samples to make the result reliable. The *i*_corr_ of the substrate slightly decreased from 45.29 ± 1.16 to 24.18 ± 1.62 μA·cm^−2^ after coating with the film. Moreover, the shape of the two polarization curves exhibit different characteristics. In the curve of the bare alloy, the current density increases sharply above *E*_corr_, indicating that *E*_corr_ is related to the pitting corrosion. In the case of the coated sample, the current density in the anodic side in the *E*_corr_ range of −1.58 to −1.51 V increases slowly, showing a corrosion inhibiting effect and protective property. However, the corrosion rate increases rapidly when potential increases above −1.50 V, indicating that the aggressive medium already permeated through the crack in the film and that pitting corrosion occurred. The EIS curves show that the Nyquist plot for the substrate only contains two loops: a capacitance loop at high frequency and an inductance loop at low frequency, which are related to the electric double layer at the interface of the substrate and solution and the pit corrosion, respectively. However, the plot obtained for the coated sample is different and consists of three loops (i.e., another medium frequency capacitance loop appears). The high frequency loop is associated with the charge transfer resistance of the film, and the other two loops may be associated with the cracks in the film, as there are many micro-cracks in Figure 1b. The |*Z*| value of the coated sample is about two times as large as that of the substrate. The equivalent circuit models for the two EIS plots are shown in Figure 6. The fitting results are listed in Table 2. *R*_s_ represents the solution resistance. *R*_t_ and *Q*_dl_ represent the charge transfer resistance and electric double layer capacity at the interface of the Mg substrate and electrolyte for Figure 6a, while representing the diffusion process of electrolytes through the cracks of the film for Figure 6b (which is actually the coating cracks resistance, *R*_crack_). *Q*_f_ and *R*_f_ represent the capacity and resistance of the film, respectively. *R*_L_ and *L* represent the inductance resistance and inductance, respectively. From Table 2, it can be seen that the *R*_f_ value is larger than *R*_t_. It can be implied that the film has a better dielectric property and charge resistance than the substrate, but the crack is the weak site.

**Figure 7:**
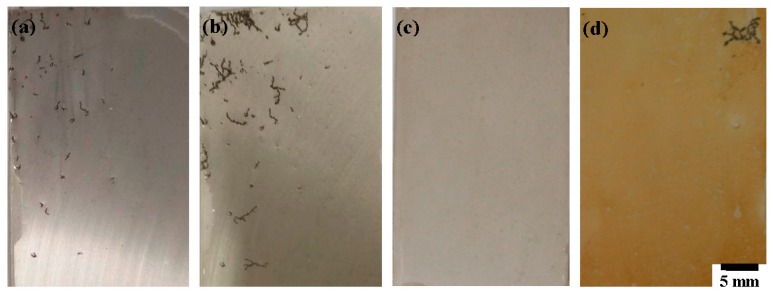
Optical morphology of (**a**,**b**) the AM60 substrate and (**c**,**d**) the sample coated with film after immersion tests for 12 h and 24 h in 0.6 M NaCl, respectively.

Additionally, an immersion test was also carried out to investigate the corrosion resistance of the film. Figure 7 shows the optical morphology of the AM60 alloy with and without film after 12 and 24 h immersion in 0.6 M NaCl solution, respectively. The macroscopic image of the substrate shows a localized (pitting and filiform) corrosion attack after 12 h immersion (Figure 7a). Conversely, no corrosion is visible on the specimen with film (Figure 7c). After 24 h, the bare alloy undergoes more serious corrosion (Figure 7b), while the majority of the coated sample is not attacked except for the limited areas of filiform corrosion (Figure 7d). However, the color of the film has changed from white gray to yellow. The above results demonstrate that this film can avoid the direct exposure of the substrate to the environment and block the penetration of the solution. As a result, the corrosion initiation time of the coated sample is delayed. However, the color alternation of the film indicates that the corrosion reaction also occurred when the coating comes into contact with a severely aggressive medium. Afterwards, once the electrolyte penetrated through the cracks and got in touch with the substrate, corrosion started from these defects. According to the electrochemical and immersion tests, the composite phosphate film can offer acceptable corrosion protection to AM60, which has the potential to act as an effective and economic anticorrosion film for Mg alloys.

## 4. Conclusions

In conclusion, a composite PCC containing Ba, Mn, Ca, and Mg has been prepared on AM60 alloy. The composition of the film has been systematically investigated. The film is composed of both amorphous and crystalline phases. The film contains Mg/Mn/Ba-HPO_4_^2^^−^ precipitates as the primary components, with some Ca/Mg/Ba-PO_4_^3^^−^ compounds and Ba–containing amorphous phases. The formation mechanism of the phosphatefilm is proposed as follows: (i) dissolution of the AM60 substrate, resulting in a large increase of the OH^−^ concentration and hydrogen evolution; (ii) reduction of H_2_PO_4_^−^ to form HPO_4_^2^^−^, a minority of which is further reduced to PO_4_^3^^−^; (iii) deposition of the more insoluble compounds, Ca/Mg/Ba-PO_4_^3^^−^; and (iv) adsorption of the abundant ions from the treating bath to precipitate the Mg/Mn/Ba-HPO_4_^2^^−^ growth of the film. This composite phosphate film can improve the corrosion resistance of the AM60 alloy, which reduces the corrosion rate of the substrate by half, as well as delays the initiation of localized corrosion. When the bare alloy undergoes serious corrosion, the coated sample is not attacked, except for the limited areas. In others words, this simple chemical conversion method to prepare a film at ambient temperatures within 0.5 h will benefit the protection of Mg alloys for applications in industries.

## Figures and Tables

**Table 1:** The content of various elements in the film tested by EDS.

Element	C	O	Mg	P	Ca	Mn	Ba	Al
Content (at %)	3.31	58.81	13.93	11.47	0.97	4.96	4.58	20.7

**Table 2:** Fitting results of the EIS spectra for the AM60 alloy with and without film immersed in 0.6 M NaCl solution.

Sample	*R*_s_ (Ω·cm^2^)	*Y*_0_ (μΩ^−1^·cm^−2^·s^−1^)	*n*	*R*_f_ (Ω·cm^2^)	*Y*_0_’ (μΩ^−1^·cm^−2^·s^−1^)	*n*’	*R*_t_ (Ω·cm^2^)	*L* (kH·cm^2^)	*R*_L_ (Ω·cm^2^)
Base	15.9	-	-	-	19.08	0.87	870	557	645
Film	19.04	10.46	0.82	1162	7.48	0.88	582	219	2519
